# Hip Arthroscopic Resection of an Intra-Articular Fibroma of the Tendon Sheath

**DOI:** 10.1155/2018/4549836

**Published:** 2018-07-29

**Authors:** Lucas Korcek, Benjamin Hoch, Dustin Richter

**Affiliations:** University of New Mexico, Albuquerque, USA

## Abstract

Fibroma of the tendon sheath most often presents around small joints and involves the tendon and tendon sheaths of the fingers, hands, and wrist. In rare instances, it presents as an intra-articular mass. It has never been described in the hip joint. In the current case presentation, this benign tumor was found to be the source of a patient's atypical severe hip pain. Arthroscopic resection of this tumor alleviated the patient's pain.

## 1. Introduction

Fibroma of the tendon sheath (FTS) is a benign fibroblastic neoplasm that most often presents as a circumscribed nodular mass around small joints and involves the tendon and tendon sheaths of the fingers, hands, and wrist [1]. In rare instances, it presents as an intra-articular mass that may have a multinodular growth pattern [2]. A sensation of fullness and pain are the most common presenting symptoms [3]. In certain instances, local bony erosion has been noted [4]. A recent review of the literature summarized the reported cases of this condition arising in and around larger joints [5]; however, to the best of our knowledge, it has never been described within the hip joint. In the current case presentation, this benign tumor was found to be the source of severe hip pain. Arthroscopic resection of this tumor alleviated the pain symptoms.

## 2. Case Presentation

A 29-year-old female presented for evaluation of progressive right hip pain over the course of several months without a known injury event. She endorsed a constant pain, feelings of tightness in the hip, and a sensation of hip stiffening with ambulation. Physical exam demonstrated significant pain and guarding with passive hip motion, particularly hip flexion past 90 degrees, and provocative maneuvers such as both flexion/adduction/internal rotation (FADIR) and flexion/abduction/external rotation (FABER), suggesting an intra-articular source of her discomfort. There was no palpable mass or neurologic deficits.

**Figure 1:**
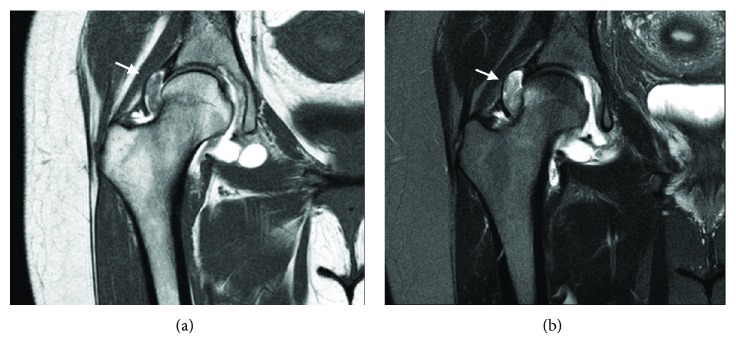
(a) T1 coronal magnetic resonance image showing nodular intra-articular mass with low signal intensity (arrow). (b) T2 fat-saturated coronal magnetic resonance image showing nodular intra-articular mass with high signal intensity (arrow).

The radiographic workup included an AP of the pelvis and special views of the right hip including Dunn lateral and a false profile. Radiographs revealed evidence of a mild mixed-type femoroacetabular impingement (FAI; acetabular crossover sign, 55 degree alpha angle, 25 degree lateral center-edge angle). There was no evidence of degenerative changes (Tönnis grade 0). Advanced imaging with a magnetic resonance arthrogram was available for review at the initial visit and demonstrated a pedunculated intra-articular mass in the superolateral aspect of the joint, near the femoral head-neck junction (Figure 1).

**Figure 2:**
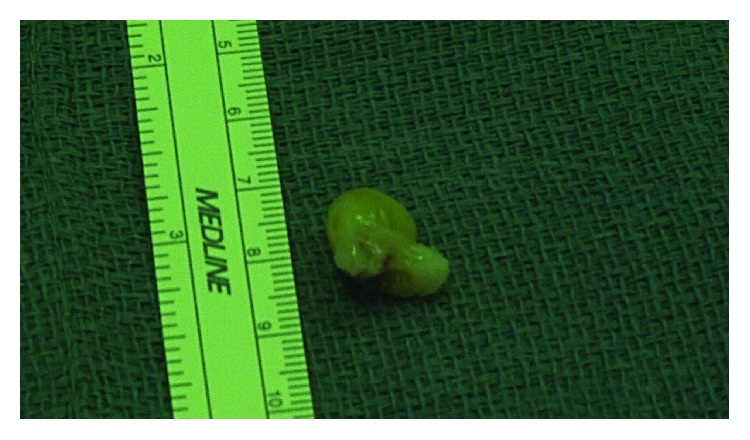
Gross specimen of the resected tumor.

Hip arthroscopy was performed in a supine position for resection of this lesion. The patient was placed in manual traction and standard midanterior and anterolateral portals were established. An extended intraportal capsulotomy was performed to allow for better access to the anterolateral femoral neck. On initial diagnostic arthroscopy, she was noted to have a concomitant anterior-superior labral tear. Minimal acetabular bone was resected and a three-anchor labral repair was performed. The traction was then released and evaluation of the peripheral compartment commenced. A nodular mass was encountered at the anterior and anterolateral femoral head-neck junction (Video 1) and excised (Figure 2). Fluoroscopic guidance was then utilized to perform a femoroplasty, and the hip capsule was closed in standard fashion.

**Figure 3:**
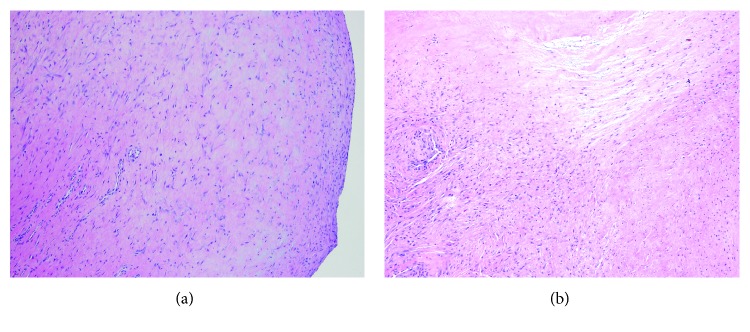
(a) Low power histopathology view demonstrating one of the circumscribed nodules that comprised the multinodular tumor. Fibroblastic cells are spindled to stellate with variable cellularity set in a collagenous stroma. Slit-like capillaries can be seen as well (center to left). The right side of the image is the surface of the nodule (magnification 10x). (b) Tumor nodules had variable cellularity with hypocellular hyalinized appearing areas (top and left) alternating with regions with higher cellularity (right) (magnification 10x).

Pathological examination of the mass was remarkable for a multinodular proliferation of benign fibroblasts with variable cellularity including hyalinized hypocellular areas (Figures 3(a) and 3(b)). The fibroblastic cells were spindle to stellate shaped with amphophilic cytoplasm and uniform oval nuclei with small nucleoli. The vascular consisted of small capillaries along with elongated slit-like vessels (Figure 3(a)). Attenuated synovium was present on the outer surface of some of the nodules. Overall, the histopathological features were characteristic of FTS; however, this case was unusual in that it had an intra-articular multinodular growth pattern akin to that seen with diffuse tenosynovial giant cell tumor involving a large joint.

Postoperatively, the patient was made 50% weight-bearing on the right lower extremity with crutches for one month. No brace was utilized. She began physical therapy the second week. At her first follow-up appointment 10 days postoperatively, she noted resolution of her hip pain and prior symptoms of “tightness.”

## 3. Discussion

FTS is defined as a benign fibroblastic nodular neoplasm that arises from the synovium of a tendon sheath. [6]. It has been suggested that FTS represents a sclerosing end stage of nodular tenosynovitis possibly associated with chromosome 2q abnormalities [7]. Though it has never been described within the hip joint, rare cases involving other large joints have been described with a similar histologic appearance to this case [2]. Suzuki et al. performed a recent literature review of case reports of FTS in and around large joints and identified 43 reported cases with the most common large joint affected being the knee (74.4%), followed by the elbow (11.6%), the ankle (9.3%), and the shoulder (4.7%). Magnetic resonance imaging (MRI) findings were described in 23 of the cases. Iso or low signal intensity of the lesion relative to skeletal muscle was seen on T1-weighted MRI. T2-weighted MRI showed various patterns. Low signal intensity of the lesion relative to the muscle was the most common; however, a high intensity signal (such as in the case presented) was seen in 4 of the 23 reports. The most common presenting symptoms were pain, swelling, and limited range of motion of the affected joint.

Arthroscopic treatment of pigmented villonodular synovitis of the hip (the suspected diagnosis prior to pathologic examination) is well described [8]. Advantages of arthroscopic over open treatment include superior visualization and access to all joint surfaces with minimal morbidity. It stands to reason that other focal intra-articular lesions are a valid indication for arthroscopic treatment as well. In the review by Suzuki and colleagues, surgical technique was described in 23/43 cases. 5 of the patients underwent arthroscopic tumor resection. Cases with tumors larger than 3 cm tended to be resected by open surgery, and there were no recurrences after surgery in either group [5].

In addition to the intra-articular mass, a very mild cam deformity was noted on our workup. While it is not possible to say if, and to what extent, this was contributing to her symptoms, the acute and rapidly worsening nature of her pain, as well as the subjectively more severe nature of her presentation than the authors typically see associated with cam impingement, lead the authors to believe the major factor in her disability was the FTS. A labral tear was identified and repaired during arthroscopy. This is a common sequelae of hip impingement [9]. The location and relative size of the fibroma compared with the relatively mild cam deformity suggest that the fibroma was a significant contributor to the pain and disability.

To our knowledge, this is the first description of FTS presenting in the hip. The patient's pain and disability was out of proportion to what is typically seen in a FAI case presentation. The case was unusual in having a multinodular intra-articular growth pattern. The described treatment with arthroscopic resection was successful in this instance.

## Supplementary Materials

Supplementary Materials Video 1: arthroscopic view of an intra-articular fibroma of tendon sheath seen from an anterolateral viewing portal in a right hip.
